# Messages From the Past: New Insights in Plant Lectin Evolution

**DOI:** 10.3389/fpls.2019.00036

**Published:** 2019-01-29

**Authors:** Sofie Van Holle, Els J. M. Van Damme

**Affiliations:** Laboratory of Biochemistry and Glycobiology, Department of Biotechnology, Ghent University, Ghent, Belgium

**Keywords:** lectin, gene family evolution, lower plants, protein domain, evolutionary diversity

## Abstract

Lectins are a large and diverse class of proteins, found in all kingdoms of life. Plants are known to express different types of carbohydrate-binding proteins, each containing at least one particular lectin domain which enables them to specifically recognize and bind carbohydrate structures. The group of plant lectins is heterogeneous in terms of structure, biological activity and function. Lectins control various aspects of plant development and defense. Some lectins facilitate recognition of exogenous danger signals or play a role in endogenous signaling pathways, while others are considered as storage proteins or involved in symbiotic relationships. In this study, we revisit the origin of the different plant lectin families in view of the recently reshaped tree of life. Due to new genomic sampling of previously unknown microbial lineages, the tree of life has expanded and was reshaped multiple times. In addition, more plant genomes especially from basal Phragmoplastophyta, bryophytes, and Salviniales (e.g., *Chara braunii, Marchantia polymorpha, Physcomitrella patens, Azolla filiculoides*, and *Salvinia cucullata*) have been analyzed, and annotated genome sequences have become accessible. We searched 38 plant genome sequences including core eudicots, monocots, gymnosperms, fern, lycophytes, bryophytes, charophytes, chlorophytes, glaucophytes, and rhodophytes for lectin motifs, performed an extensive comparative analysis of lectin domain architectures, and determined the phylogenetic and evolutionary history of lectins in the plant lineage. In conclusion, we describe the conservation of particular domains in plant lectin sequences obtained from algae to higher plants. The strong conservation of several lectin motifs highlights their significance for plants.

## Introduction

After the discovery of the Archaea, a model was proposed that divides cellular life into three evolutionary domains: “Eukarya,” “Bacteria,” and “Archaea” (Woese et al., 1990). In this three-domain tree of life, Archaea and Eukarya are sister groups that share a common ancestor. Over the years, the evolutionary relationships between Archaea and Eukarya have been the subject of long-lasting debates (Williams et al., 2013; Forterre, 2015; Hug et al., 2016). Recent comprehension of novel archaeal superphyla through metagenomic analyses and advances in molecular phylogenetics provided a novel view on the origin and early evolution of eukaryotes. Today, the two-domain topology is generally accepted, with Bacteria and Archaea being the two primary branches, in which eukaryotes have emerged from within the Archaea. Taking into account the most recent phylogenomic analyses, eukaryotes most probably originate from within the Asgard (being the closest prokaryotic relatives of eukaryotes) and not the ‘TACK’ (which groups Thaumarchaeota, Aigarchaeota, Crenarchaeota, and Korarchaeota) superphylum within the Archaea (Eme et al., 2017; Zaremba-Niedzwiedzka et al., 2017). In addition, phylotranscriptomic data show that approximately 450–500 million years ago, land plants evolved from a streptophyte algae lineage (Zygnematophyceae) (Wickett et al., 2014). The transition from unicellular and filamentous algae to modern land plants required distinctive adaptations/exaptations to the terrestrial environment including three-dimensional growth, sporophyte dominance, development of vasculature and desiccation-tolerant seeds (Harrison, 2017; Rensing, 2018; de Vries and Archibald, 2018).

Lectins are a group of diverse proteins that occur ubiquitously in nature and share the ability to recognize and bind specific carbohydrate structures. Plants lectins are mainly involved in plant immunity and symbiosis, but roles in plant development have also been attributed to particular lectins (reviewed by Van Holle and Van Damme, 2018). For a long time, most research aimed at the biochemical and functional characterization of plant lectins, while their relevance in the colonization of land by plants and in the evolution of angiosperms was neglected. Today, various studies report on the abundance of lectin genes in modern plant models and homologs of plant lectins have also been reported outside the plant kingdom (Naganuma et al., 2014; Wong et al., 2014). A recent study on lectin sequences in model species (*Arabidopsis*, rice, soybean, and cucumber) points to a dynamic evolution of these protein families (Van Holle et al., 2017). Unfortunately, only angiosperm genomes were included in these analyses, which makes it difficult to reconstruct how plant lectins diverged from their common ancestor. In 2018, the first genomic data from ferns (*Azolla filiculoides* and *Salvinia cucullata*), a close sister group to angiosperms, was published (Li et al., 2018). Furthermore, a new chromosome-scale assembly of the *Physcomitrella patens* genome, a model for the mosses, was first released in 2017 (Lang et al., 2018). The genome sequences of *Marchantia polymorpha*, a model species for the liverwort lineage, is believed to represent the last common ancestor of extant land plants the best because of its low genetic redundancy (Bowman et al., 2017). However, the true bryophyte topology is still enigmatic and the relevance of the liverwort *Marchantia polymorpha* as a model for the earliest land plants is heavily disputed (Puttick et al., 2018; Rensing, 2018). The genome of *Chara braunii* (Nishiyama et al., 2018) and of *Klebsormidium nitens* (Hori et al., 2014) represent the Charophyceae and Klebsormidiophyceae, charophycean algae that share a common ancestor with land plants. In addition to these key genomes of the streptophyte lineage, the study of genome sequences from Chlorophyta (including prasinophytes and core chlorophytes), the freshwater microscopic algae *Cyanophora paradoxa* (Price et al., 2012) and rhodophytes (*Cyanidioschyzon merolae* and *Porphyra umbilicalis*) (Matsuzaki et al., 2004; Brawley et al., 2017) can further refine the divergence of the plant lectin family and their establishment during land plant evolution.

Since substantial progress has been made recently in resolving the placement of eukaryotes within the Archaea, the primary focus of our study relates to the origin of plant lectins in the tree of life. We attempted to reconstruct the evolutionary origins of the plant lectin families. Our data highlight that some families are a eukaryotic innovation, while others are descendants of ancient protein families as they are also found in prokaryotes. We also considered the domain architectures and diversification of specific lectin families, with emphasis on the similarities/differences between land plant lineages and in lineages that are sister to land plants.

## Materials and Methods

### Assembly of Dataset of Plant Lectin Homologs in the Tree of Life

**FIGURE 1:**
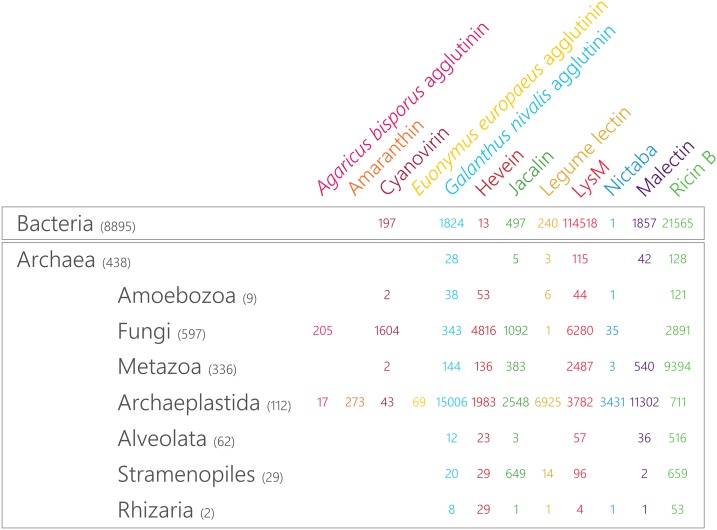
Taxonomic distribution and magnitude of plant lectin domains in the tree of life. The size of the lectin domain families is retrieved from Interpro 71.0 and the number of included reference proteomes in each of the lineages is indicated between brackets.

In this study, eleven plant lectin families (Van Damme et al., 1998) [represented by *Agaricus bisporus* agglutinin, amaranthin, cyanovirin, *Euonymus*-related lectin (EUL), *Galanthus nivalis* lectin (GNA), hevein, jacalin, legume lectin, LysM: lysin motif, *Nicotiana tabacum* agglutinin (Nictaba) and ricin B], together with the malectin family -initially reported in Metazoa (Schallus et al., 2008)- have been considered. The availability of a unique Pfam and Interpro identifier for each lectin family facilitated a straightforward search for lectin sequences in a large dataset. In total, 38 plant genomes were selected, representing diverse clades of Archaeplastida, including 15 core eudicots (*Arabidopsis thaliana, Capsella rubella, Brassica rapa, Theobroma cacao, Citrus clementina, Populus trichocarpa, Linum usitatissimum, Ricinus communis, Malus domestica, Cucumis sativus, Glycine max, Medicago truncatula, Vitis vinifera, Solanum lycopersicum*, and *Amaranthus hypochondriacus*), five monocots (*Zea mays, Sorghum bicolor, Brachypodium distachyon, Oryza sativa*, and *Musa acuminata*), one basal angiosperm (*Amborella trichopoda*), two gymnosperms (*Ginkgo biloba* and *Picea abies*), two Polypodiopsida (*Azolla filiculoides* and *Salvinia cucullata*), one lycophyte (*Selaginella moellendorffii*), three bryophytes (*Physcomitrella patens, Sphagnum fallax* and *Marchantia polymorpha*), one Charophyceae (*Chara braunii*), one Klebsormidiophyceae (*Klebsormidium nitens*), two core chlorophytes (*Chlorella* NC64A and *Chlamydomonas reinhardtii*), two prasinophytes (*Ostreococcus lucimarinus* and *Micromonas* sp. RCC299), one glaucophyte (*Cyanophora paradoxa*) and two rhodophytes (*Cyanidioschyzon merolae* and *Porphyra umbilicalis*). Exploration of the plant comparative genomics platform PLAZA 4.0^1^ (Van Bel et al., 2018) employing the Interpro identifier revealed the abundance of lectin domains. Following Interpro identifiers were used: IPR009960 (*Agaricus bisporus* agglutinin), IPR008998 (amaranthin), IPR011058 (cyanovirin), IPR001480 (GNA), IPR001002 (hevein), IPR001229 (jacalin), IPR001220 (legume lectin), IPR018392 (LysM), IPR024788 and IPR021720 (malectin), IPR025886 (Nictaba), and IPR000772 (ricin B). The domain architecture of the retrieved sequences was analyzed using the Interpro, incorporated in the PLAZA 4.0 toolbox. Because EUL and ricin B lectin domains are characterized by the same identifier, the extent of the EUL family was estimated based on the results from Fouquaert et al. (2009a) and De Schutter et al. (2017). Additionally, Gymno PLAZA 1.0^2^ (Proost et al., 2015) and Phytozome v12.1.5^3^ (Goodstein et al., 2012) were employed to investigate the distribution of lectin sequences in *Ginkgo biloba* and *Musa acuminata, Linum usitatissimum, Sorghum bicolor, Brachypodium distachyon, Sphagnum fallax, Ostreococcus lucimarinus, Micromonas* sp. RCC299, and *Porphyra umbilicalis* using a similar approach. The Interpro or Pfam identifier was used to search for sequences accommodating the lectin motifs and the amino acid sequences were downloaded. Next, the sequences were subjected to analysis with Interpro 71.0 at https://www.ebi.ac.uk/interpro/ (Mitchell et al., 2015) to explore the domain architecture. Putative lectin sequences from *Cyanidioschyzon merolae, Cyanophora paradoxa, Chlorella* NC64A, *Klebsormidium nitens, Chara braunii, Azolla filiculoides*, and *Salvinia cucullata* were identified through BLASTp searches online^4^^,^^5^^,^^6^^,^^7^^,^^8^^,^^9^ using a representative query for each of the lectin families. Using the following settings, expect value: 10 and substitution matrix: BLOSUM62, the best hit was used for a consecutive BLASTp search to obtain all possible lectin sequences. Next, the presence of lectin domains (and other protein domains) was verified with Interpro 71.0 at https://www.ebi.ac.uk/interpro/ (Mitchell et al., 2015). Supplementary Table 1 compiles the obtained results of this large genome-wide search for lectin motifs. The Interpro database (71.0) was used to estimate the occurrence of lectin domains in different lineages in the tree of life. The results are compiled in Figure 1 and represent estimates of the size of the different lectin families.

### Evolutionary Expansion of Plant Lectin Genes

To understand the evolution of the hevein, jacalin and Nictaba gene family, reconciliation trees were generated using Notung 2.9 (Stolzer et al., 2012). Trees were rooted in Notung and in the reconciliation mode, the edge weight threshold was set to 0.0005, the loss cost to 1, the duplication to 1.5 and the co-divergence cost to 0. The species tree was constructed in NCBI Taxonomy Common Tree^10^ and contained the following species: *Amaranthus hypochondriacus, Amborella trichopoda, Arabidopsis thaliana, Azolla filiculoides, Brassica rapa, Capsella rubella, Chara braunii, Chlamydomonas reinhardtii, Chlorella* NC64A, *Cucumis sativus, Cyanidioschyzon merolae, Cyanophora paradoxa, Ginkgo biloba, Glycine max, Klebsormidium nitens, Linum usitatissimum, Malus domestica, Marchantia polymorpha, Micromonas* sp. RCC299, *Musa acuminata, Oryza sativa, Picea abies, Physcomitrella patens, Populus trichocarpa, Porphyra umbilicalis, Salvinia cucullata, Selaginella moellendorffii, Solanum lycopersicum, Sorghum bicolor, Sphagnum fallax, Theobroma cacao, Vitis vinifera*. Amino acid sequences for hevein, jacalin and Nictaba homologs present in these species were downloaded from the Phytozome 12.1 database (see footnote 3), PLAZA 4.0 (see footnote 1), Gymno PLAZA 1.0^2^ (see footnotes 2, 6, 7, 8, 9). Multiple sequence alignments were performed online with MAFFT version 7^11^ using default settings (Katoh et al., 2017). Subsequently, the aligned sequences were subjected to trimAl v.3 (Capella-Gutiérrez et al., 2009) to trim the alignments to the gap threshold of “gt 0.6” and remove all columns with gaps in more than 40% of the sequences. Next, maximum likelihood gene trees were constructed with IQ-TREE 1.3.1 [automated selection of the substitution model including FreeRate heterogeneity, ultrafast bootstrap approximation (UFBoot) with 1,000 bootstrap alignments] (Nguyen et al., 2015; Kalyaanamoorthy et al., 2017; Hoang et al., 2018). Whole genome duplication and triplication events as described by Li et al. (2016, 2018) and Lang et al. (2018) were added to the gene trees reconciled with the species tree. Figtree v. 1.4.3 was used to visualize and modify the phylogenetic trees^12^.

### Sequence Motif Analysis

Sequence motif analysis was performed online with MEME suite 5.0.0^13^ (Bailey et al., 2009). Protein datasets were mined for conserved motifs within the lectin domain sequences that were identified in the 38 plant species described above. Parameters were set as follows: classic mode, window size of 6–50. The distribution of selected significant motifs was analyzed across sequences and species.

## Results and Discussion

### Evidence for Plant Lectin Domains in Bacteria and Archaea

In the plant kingdom, several unique lectin families have been reported and each of them is defined by a characteristic carbohydrate-recognition or lectin domain (Van Damme et al., 2008). Taking advantage of the wealth of available sequenced genomes, we mined the predicted proteomes of all species available in the Interpro 71.0 database for plant lectin motifs. As shown in Figure 1, the occurrence of most plant lectin domains is not restricted to the plant kingdom. While the distribution of the amaranthins and the EUL family is limited to plants, all other lectin domains are also present in other lineages of the tree of life. However, large differences in the number of sequences within one particular family are observed between the different lineages (Figure 1). Furthermore, the discrepancy between lectin families points to distinct evolutionary paths. The malectin family is represented by two Interpro identifiers: the “Malectin domain” (IPR021720) and the “Malectin-like domain” (IPR024788). In Figure 1, the combined number of sequences for both identifiers is shown. It should be mentioned that the “Malectin-like domain” (IPR024788) could only be retrieved in Archaeplastida (with 7,771 hits) and in none of the other lineages.

### Not All Plant Lectin Domains Originate From Within the Archaeplastida

**FIGURE 2:**
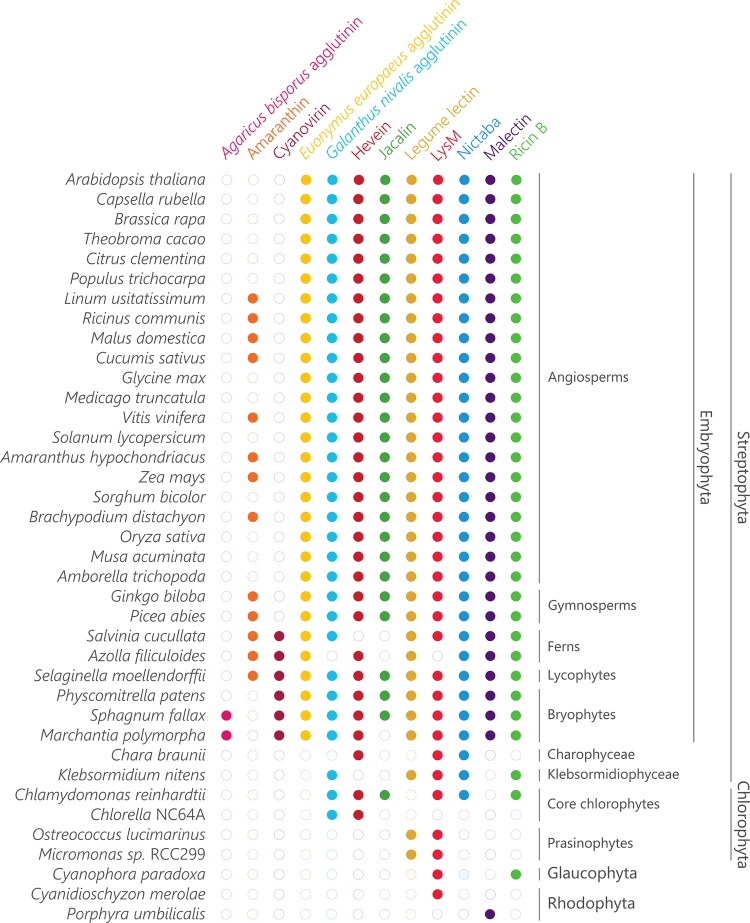
Sampling of plant lectin domains from the plant lineage. Coulson plot demonstrating presence or absence of genes encoding plant lectins across a range of Archaeplastida. Filled circles indicate genes identified with high confidence.

To reconstruct the evolutionary paths for the different plant lectin domains, representative genomes of the most important linages within Archaeplastida (including core eudicots, monocots, one basal angiosperm, two gymnosperms, two ferns, one lycophyte, three bryophytes, one Charophyceae, one Klebsormidiophyceae, two core chlorophytes, two prasinophytes, one glaucophyte and two rhodophytes) were screened for the presence of plant lectin domains (Figure 2). It is clear that the distribution of the lectin motifs is variable, pointing toward a different evolutionary origin for different lectin domains.

**FIGURE 3:**
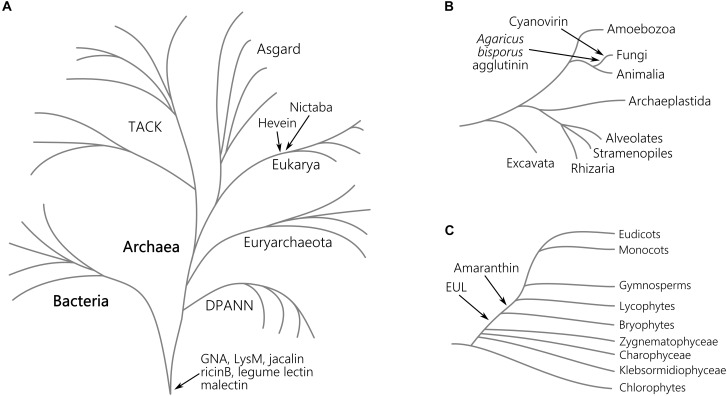
Origin of lectin motifs in the tree of the life. Schematic representation of the major clades of the tree of life evolved from the last common ancestor **(A)**, the eukaryotic lineage **(B),** and the Viridiplantae **(C)**. For each plant lectin domain, the presumed origin is indicated. The depicted tree of life is based on the current genomic sampling of life described by Eme et al. (2017).

The *Agaricus bisporus* agglutinin originates from fungi and data shown in Figure 1 confirm that this lineage encompasses the highest number of *Agaricus bisporus* agglutinin homologs. In plants, the presence of this lectin domain is restricted to the bryophytes (Figure 2). Homologs of the *Agaricus bisporus* agglutinin were only retrieved from the genomes of the liverwort *Marchantia polymorpha* and the bog moss *Sphagnum fallax*. This is the first record of a homolog for the *Agaricus bisporus* agglutinin in mosses, as functional lectins of this family have only been described in fungi and *Marchantia polymorpha* (Peumans et al., 2007; Bovi et al., 2011). It can be assumed that this lectin domain arose in a fungal ancestor (Figure 3) and that horizontal gene transfer, possibly through endosymbionts, is responsible for its confined existence in Archaeplastida.

A similar story holds for the cyanovirin lectin domain. This lectin domain is mostly present in fungi (Figure 1) and to a lesser degree in Bacteria, Amoebozoa, Metazoa, and plants. The rather limited distribution in Bacteria and Eukarya points to multiple independent horizontal gene transfers between fungi and Bacteria, and/or between fungi and an ancestor of Embryophyta (Figure 3) as suggested earlier (Percudani et al., 2005). Clearly, the cyanovirin domain was purged during the evolution of gymnosperms and angiosperms (Figure 2).

The occurrence of the amaranthin domain is limited to vascular plants (lycophytes, ferns, gymnosperms and angiosperms) and scattered over different families but is certainly not ubiquitous (Figure 2). This taxonomic distribution pattern is very mystifying which makes it difficult to reveal the exact phylogeny, but suggests an origin within the vascular plant lineage (Figure 3). This is in line with a recent study, in which amaranthin sequences were identified in 33 plant genomes. Here, a similar complex distribution pattern was also observed (Dang et al., 2017).

Similar to the amaranthins, the EUL family represents a true plant lectin family. EUL homologs are found in land plants (Embryophyta) including the bryophyte lineage, but unlike the amaranthins, are omnipresent. Presumably, this protein domain arose in the last common ancestor of the Embryophyta (Figure 3) and remained part of the lectin collection during the development of modern land plants. The results validate an earlier study in which the complete genome sequence of *Marchantia polymorpha* was not yet available (Fouquaert et al., 2009a). A striking correlation was observed between the origin of the EUL family and the occurrence of stomata. Ancient types of stomata are described in members of the bryophyte lineage (Chater et al., 2017) while homologs of ArathEULS3, a lectin involved in stomatal closure (Van Hove et al., 2015), originate from the same lineage. Deciphering the function of the EUL homologs and other lectins in these extant plant species will bring clarity into their ancestral role, furthering their evolutionary history and explain how they evolved to a diversified group of proteins in higher plants. This could help us to answer the question whether the evolution of the EUL family occurred in parallel with the stomatal development during terrestrial transition of plants, and will be important to elucidate their function.

The distribution of GNA, LysM, jacalin, ricin B, legume lectin and malectin domains in all lineages of the tree of life (Figure 1) proposes an origin in the last universal common ancestor of Bacteria and Archaea (Figure 3). The GNA, jacalin, malectin and legume lectin sequences are more prevalent in plants while LysM and ricin B domains are most abundant in Bacteria (Figure 1). Malectin domains could only be retrieved from embryophyte genomes and the rhodophyte *Porphyra umbilicalis*, while GNA, LysM, ricin B and legume lectins homologs appear to be ubiquitous in Viridiplantae, including Chlorophyta and Streptophyta (Figure 2). In the glaucophyte *Cyanophora paradoxa*, only LysM and ricin B lectin domains could be retrieved. LysM lectin domains were also identified in one of the two rhodophyte species under study, *Cyanidioschyzon merolae*. Especially for the jacalin and LysM lectin family, several studies have already reported on their widespread distribution (Nagata et al., 2005; Buist et al., 2008; Zhang et al., 2009; Kanagawa et al., 2014; Naganuma et al., 2014; Akcapinar et al., 2015).

Judging from Figure 1, hevein and Nictaba-related lectins are a eukaryotic innovation. Because the hevein domain is shared by the most significant eukaryotic lineages (Stramenopiles, Rhizaria, Alveolata, Amoebozoa, Archaeplastida, Fungi, and Metazoa), it must have arisen in their last common ancestor, and evolved independently after the lineages split (Figure 3). Our data show that the number of hevein sequences varies considerably with more than 1,900 homologs in fungi and Archaeplastida (Viridiplantae, Glaucophyta, and Rhodophyta), 136 sequences in Metazoa (animals) and less than 54 homologs in the other clades. The hevein domain has also been reported in some nematode species (Bauters et al., 2017). The hevein domain is absent from Archaea, but 13 homologs were mined from Bacteria, in particular in Burkholderiales and Brenneria, containing mostly plant pathogenic bacteria (Ura et al., 2006; Young and Park, 2007; Maes et al., 2009; Ham et al., 2011; Lee et al., 2016). The Nictaba domain on the other hand, is more confined to Archaeplastida and fungi since three hits were found in Metazoa and only one hit in Rhizaria, Amoebozoa, and Bacteria. Differential loss of homologous genes in the genomes of Amoebozoa, Rhizaria, and Metazoa, rather than multiple independent horizontal gene transfers possibly account for the complex pattern of Nictaba sequences in Eukaryota. In the green lineage, both Nictaba and hevein sequences are widespread and present in land plants and core chlorophytes. In Klebsormidiophyceae, only Nictaba sequences are found while Charophyceae contain hevein homologs in addition to Nictaba lectins (Figure 2).

### Lectin Homologs Are Variably Maintained Across a Broad Range of Plant Lineages

Plant lectin genes were exploited in key genomes of significant lineages in Archaeplastida. The comparative analysis of the lectin sequences retrieved from the rhodophytes *Cyanidioschyzon merolae* and *Porphyra umbilicalis*; the glaucophyte *Cyanophora paradoxa*; chlorophytes *Micromonas* sp. RCC299, *Chlorella* NC64A, *Chlamydomonas reinhardtii*; Klebsormidiophyceae *Klebsormidium nitens*; Charophyceae *Chara braunii*; bryophytes *Marchantia polymorpha, Physcomitrella patens*; Polypodiopsida *Azolla filiculoides, Salvinia cucullata* and gymnosperms *Picea abies* and *Ginkgo biloba*; revealed a large discrepancy in the organization and distribution of the lectin families (Supplementary Tables 1, 2 and Figure 2). Representatives of the *Agaricus bisporus* agglutinin are confined to the genomes of *Marchantia polymorpha* and *Sphagnum fallax* while amaranthins are not yet present in this plant lineage. The earliest records of amaranthin homologs are found in lycophytes. EUL homologs are found in bryophytes and vascular plants and represent a rather small family, except in the *Ginkgo biloba* genome. Similarly, the cyanovirin family present in bryophytes, lycophytes and ferns only represents a small fraction of the lectin collection in these species. Remarkably, it is the second largest lectin family (22.4%) in *Salvinia cucullata*. GNA homologs appear as a large fraction of the total number of lectin genes in *Marchantia polymorpha, Physcomitrella patens* and *Picea abies* but are a rather small family in *Chlamydomonas reinhardtii, Klebsormidium nitens*, and *Ginkgo biloba*. The size of the hevein family is very much dependent on the plant species, analogously to GNA homologs. Lectin homologs belonging to the jacalin and LysM family represent more than 70% of the total number of lectin genes in the chlorophyte *Chlamydomonas reinhardtii*, whereas no jacalin-related lectins could be retrieved from most other plant species. Legume lectin sequences account for the largest lectin families in *Marchantia polymorpha, Azolla filiculoides, Salvinia cucullata*, and *Picea abies*. Only one or two lectin motifs are identified in *Cyanophora paradoxa* (LysM), *Micromonas* sp. RCC299 (legume lectin, LysM), *Chlorella* NC64A (GNA, hevein) and *Cyanidioschyzon merolae* (LysM) while only malectins were retrieved from the genome of *Porphyra umbilicalis*. Clearly, there is no evidence for widespread or abundant lectin motifs in prasinophytes, glaucophytes, and rhodophytes.

### Diversification of Domain Arrangements in Higher Plant Lineages

**FIGURE 4:**
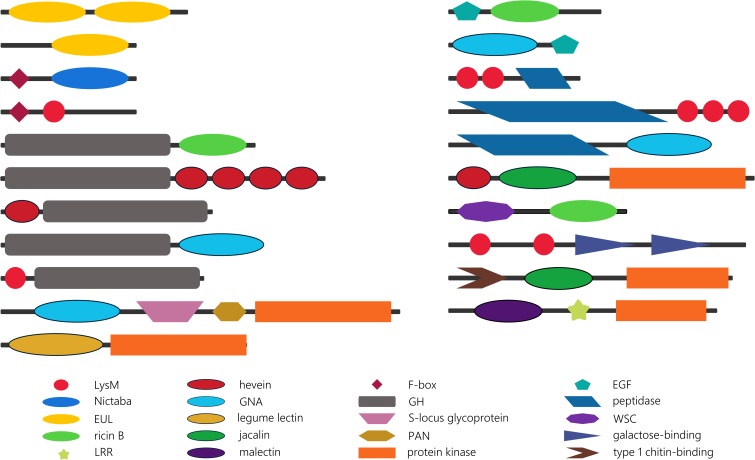
Schematic overview of some striking lectin domain architectures.

To gain more insight into their evolutionary history, the domain organization of the lectin sequences was investigated. The presence of multi-domain proteins in the genomes of all kingdoms of life has been reported before (Ekman et al., 2005). As a result of their more complex genome and biology, higher eukaryotes are considered to display a larger collection of multi-domain proteins (Ekman et al., 2007). Supplementary Table 3 summarizes the domain architectures of plant lectin sequences in 14 rhodophytes, glaucophytes, chlorophytes, Klebsormidiophyceae, Charophyceae, bryophytes, lycophytes, Polypodiopsida and gymnosperms, and the preservation of these domain architectures in four core model angiosperms (*Arabidopsis thaliana, Glycine max, Cucumis sativus*, and *Oryza sativa*). A comprehensive analysis of all the protein domains associated with plant lectin domains in the plant species under study (*Cyanidioschyzon merolae, Porphyra umbilicalis, Cyanophora paradoxa, Micromonas* sp. RCC299, *Chlorella* NC64A, *Chlamydomonas reinhardtii, Klebsormidium nitens, Chara braunii, Marchantia polymorpha, Physcomitrella patens, Azolla filiculoides, Salvinia cucullata, Picea abies*, and *Ginkgo biloba*) yielded an extensive list of protein domain arrangements (Supplementary Table 3). The description of each protein domain and lectin domain combination is beyond the scope of this manuscript. Below we describe some striking observations and interesting domain combinations (Figure 4).

The EUL family groups all sequences with an *Euonymus*-related lectin domain. Though the EUL lectin domain can be preceded or followed by sequences longer than 100 amino acids, no protein domains other than the lectin domain are recognized in the EUL sequences. This characteristic is unique for the EUL family. Two types of EUL domain architectures have been described: proteins consisting of two tandem arrayed EUL domains and single EUL domain proteins (Fouquaert et al., 2009a). Both single and double EUL domain proteins are present in the genomes of *Marchantia polymorpha* and *Physcomitrella patens* (Supplementary Table 3). This trait is shared with monocot lineages, while genomes from dicot species exclusively harbor single EUL domain architectures (De Schutter et al., 2017). Similar to the core eudicot genomes, single EUL domain proteins were identified in the genomes from the Polypodiopsida and the gymnosperms under study. Until now, it remains unclear why the Eudicotyledones did not maintain the double EUL domain architecture in their genome.

Unlike the EUL lectin family, sequences from all other plant lectin families are composed of lectin domains linked with a variety of other annotated protein domains. Though most lectin sequences are composed of two protein domains, lectin sequences with up to six different protein domains have been reported (Van Holle et al., 2017). Analysis of the identified domain architectures in the lectin sequences retrieved from the genomes that are sister to vascular plants, land plants, Streptophyta and Viridiplantae under study, revealed some remarkable similarities with the domain organization of lectin sequences in higher plants. Similar to the model species *Arabidopsis thaliana, Oryza sativa, Glycine max* and *Cucumis sativus*; glycoside hydrolase (GH), protein kinase or F-box domains are often found in combination with lectin domains in sisters of Streptophyta and vascular plants. It could be postulated that most of these domain architectures arose in a common ancestor of Streptophyta and/or chlorophytes, and that have been retained during evolution. However, there are a number of anomalies. The combination of an F-box domain with a Nictaba or LysM domain is shared by all Viridiplantae, while the F-box/jacalin combination is only present in higher plants (Van Holle et al., 2017). Similarly protein architectures involving GH and hevein or ricin domains are ubiquitous in Viridiplantae while the association of a GH domain and a GNA or LysM domain in the genomes under study is restricted to bryophytes. Moreover, the combination of GH and hevein domains is determinative in terms of the order of the domains, and in terms of the type of GH. In the PLAZA 4.0 database, hevein/GH sequences are defined as Embryophyta-specific, while GH/hevein domain organization is Chlorophyta-specific. Furthermore, the hevein/GH18 domain combination is only present in bryophytes; in contrast to the hevein/GH19 combination which is shared by Embryophyta (Supplementary Table 3). Previous studies reported on the expansion of the receptor-like protein kinases in the plant lineage (Lehti-Shiu and Shiu, 2012; Xing et al., 2013) and suggest that receptor-like protein kinases originated within the Streptophyta lineage, with a significant increase in gene number in angiosperms. Embryophyta are the earliest lineage in which lectin receptor-like protein kinases are found, as illustrated in the GNA family. The GNA/S-locus glycoprotein/PAN/protein kinase domain combination is found twice in the genome of *Physcomitrella patens* but up to 76 and 86 sequences with the same architecture are present in the genome of *Oryza sativa* and *Glycine max*, respectively. The expansion of legume/protein kinase sequences evolved in a similar way, but there is also a very large set of homologous sequences in *Marchantia polymorpha* (Supplementary Table 3). The first record of a lectin receptor-like protein kinase (LysM-RLK) was reported in the *Chara braunii* genome, and recently confirmed by the work of Nishiyama et al. (2018). No lectin receptor-like protein kinases were retrieved from the chlorophytes nor in more distant lineages (glaucophytes or rhodophytes). However, the combination of the LysM domain and a protein kinase domain is also present in bacteria (e.g., UniProt A0A0N0UXM0; A1ZLP4), suggesting that these identical domain architectures arose independently in different lineages.

The lectin sequences retrieved from sisters of angiosperms and from algae revealed some new domain combinations that are only present in rhodophytes, glaucophytes, chlorophytes, charophytes, bryophytes, ferns and/or gymnosperms. Several peptidase domains and the epidermal growth factor (EGF)-like domain are examples of protein domains that are not found in association with lectin domains in angiosperms (Supplementary Table 3). The EGF domain might not be common in the plant kingdom, but is also present in animal lectins. In particular in C-type lectins, the EGF domain is associated with the C-type lectin domain in many different domain arrangements. Some of them include the combination of a C-type lectin domain with multiple EGF domains, sometimes in combination with other protein domains. In vertebrates, C-type lectins have numerous functions, the most important being key players in pathogen sensing and the initiation of immune responses (Mayer et al., 2017; Xia et al., 2018). In general, proteins with EGF domains are predominantly found in a large number of animal protein sequences (Zeng and Harris, 2014). In our analysis, combinations of the EGF-like domain with the GNA and ricin B lectin domains were identified in chlorophytes and/or bryophytes. Thus, it can be postulated that the presence of EGF/lectin domain combinations in these species originates from a eukaryotic lineage, the ancestor of both the plant and animal lineage. The EGF-like domain was originally preserved in chlorophytes and bryophytes, but was subsequently eliminated from the gene set in modern plants.

Peptidase M23 and peptidase C1A domains are associated with LysM domains and the peptidase M11 domain is found in combination with the GNA domain. These examples illustrate the range of specificities of the peptidase domains. The M11 peptidase is a metalloprotease from *Chlamydomonas reinhardtii* that is involved in cell wall degradation. Next to *Chlamydomonas reinhardtii*, it was also reported in *Volvox carteri* (Kubo et al., 2002). The M23 peptidase has a bacterial origin, similar to the LysM domain to which it is associated. In Archaeplastida, combinations of the M23 peptidase domain and LysM domain were identified in glaucophyta, charophyta, and bryophytes. All these sequences contain multiple LysM domains. In contrast to the M23 peptidases, the C1A peptidases represent mainly a eukaryotic family, with homologs in both the plant and animal kingdom (Santamaría et al., 2014). Although this protein domain is widespread in Viridiplantae, sequences involving a combination of the C1A peptidase and lectin domains have not been retained in vascular plants.

Another striking observation is the unique combination of two different lectin domains (in particular a hevein domain and a jacalin domain, a LysM domain and the fucolectin tachylectin-4 pentraxin-1 domain, and a LysM domain and two C-type lectin domains) in *Chlamydomonas reinhardtii*. In Nematoda, sequences involving a hevein domain and multiple LysM domains were previously reported (Bauters et al., 2017). Furthermore, domain architectures in which both a lectin domain (ricin B, LysM or jacalin) and at least one other sugar-binding domain (carbohydrate-binding WSC, galactose-binding domain, type 1 chitin-binding domain) have been identified (Supplementary Table 3). In the latter case, the lectin domain and the additional carbohydrate-binding domain most probably display a different carbohydrate-binding specificity. It should be mentioned that further studies at protein level are needed to investigate the functionality of the domains, since the carbohydrate-binding activity of lectin domains cannot be guaranteed based on the presence of a protein sequence.

Regarding the domain arrangement of lectin sequences in basal plant lineages, the multitude of sequences with tandem arrayed lectin domains is noteworthy. Sequences with a two or three LysM domains (in combination with a protein kinase domain) are conserved throughout Archaeplastida. In Arabidopsis and rice, they were identified as part of the plant immune system where they play key roles in the perception and recognition of danger signals. Similar proteins in legumes facilitate symbiotic communication (Zipfel and Oldroyd, 2017; Van Holle and Van Damme, 2018). A sequence composed of four hevein domains was already described in rice (Van Holle et al., 2017), but domain architectures involving more than two hevein domains and additional protein kinase or GH domains appear to be specific to ferns, Chlorophyta or Marchantiaceae.

### Genomic Evolution and Expansion of Nictaba, Jacalin, and Hevein Lectins

Investigation of the expansion of the lectin families during the course of evolution can be linked to specific adaptive speciation events. Three plant lectin families that are present in both land plants and chlorophytes were selected for detailed analysis. To study the genomic evolution and expansion, the Nictaba, jacalin and hevein gene trees were reconciled with a species tree, including 29 plant genomes (Supplementary Figure 1). The full reconciliation of the Nictaba, jacalin and hevein family trees with the species tree are illustrated in Supplementary Figures 2–4. The Nictaba, jacalin and hevein gene trees are shown in Supplementary Figures 5–7. Supplementary Table 4 summarizes the number of duplications, co-divergences and losses within each of the species, families and ranks.

The Nictaba family evolved through 349 duplications and 314 losses, whereas the jacalin family underwent 287 duplications and 316 losses. In contrast, during the evolution of the hevein family (the smallest in gene size), gene losses were far more abundant (370) than duplication events (216). Whole genome duplication and triplication events have been added to the species tree in Supplementary Figure 1 and are generally believed to play an important role in the expansion of gene families (Soltis et al., 2015; Panchy et al., 2016; Soltis and Soltis, 2016; Van de Peer et al., 2017). Indeed, the two duplication events that are shared by all Brassicaceae resulted in high duplication numbers for all lectin families, yet the *Brassica rapa*-specific whole genome triplication event only contributes to a high number of duplications and losses in the jacalin family. A recent study showed that the *Physcomitrella patens* genome was subjected to two rounds of whole genome duplications. There is evidence that these events are common for mosses while they were not detected in the liverwort (*Marchantia polymorpha*) and hornwort lineages (Lang et al., 2018). None of the lectin genes identified in *Physcomitrella patens* were present in the ancestral karyotype and this is also reflected by the relatively low numbers of duplication and losses for *Physcomitrella patens* (Supplementary Table 4). Overall, the expansion of the jacalin and hevein family in bryophytes is more pronounced compared to the Nictaba family. On the other hand, the latter lectin family displays a larger number of losses in fabids, accompanied by many duplications in all species of this clade. Regarding the evolution of the hevein lectin family, high duplication rates were observed in *Marchantia polymorpha, Picea abies, Amaranthus hypochondriacus, Solanum lycopersicum* and *Populus trichocarpa*. The most important duplication events for the jacalin family are assigned to *Brassica rapa, Musa acuminata, Oryza sativa, Sphagnum fallax* and *Selaginella moellendorffii*.

A large-scale study on gene duplicability across angiosperms revealed that gene duplicability is a non-random process and that most gene families are either primarily single-copy genes or multi-copy genes. Single-copy genes are related to basic cellular functions (organelle function, genome stability maintenance) whereas multi-copy genes are biased toward signaling, transport, metabolism and other cellular and biochemical functions or in other words, environmentally responsive genes (Li et al., 2016). The extended repertoire of almost all plant lectin genes in higher plants (Van Holle et al., 2017) suggests that these are multi-copy genes. However, it remains to be investigated whether there is a direct correlation between gene function and gene duplicability, and how lectin genes have contributed to the adaptation of plants in a changing environment. In a recent study focusing on the immune response of *Arabidopsis* upon recognition of bacterial flagellin, the resilience of the plant immune system was explained by network buffering (Hillmer et al., 2017). Interactions among sectors of the network provide a basis for network buffering and can successfully compensate for the loss of a single component (Hillmer et al., 2017; Tyler, 2017). Since several plant lectins are reported to be involved in plant signaling (Xiang et al., 2011; Choi et al., 2014; Ranf et al., 2015; Couto and Zipfel, 2016; Balagué et al., 2017; Erwig et al., 2017; Xu et al., 2017), and given the strong expansion of lectin genes, partially as a result of polyploidization, homologous lectins are suggested to have subfunctionalized and could potentially facilitate network buffering in angiosperms.

### Conserved Motifs in the Nictaba, Jacalin, and Hevein Lectin Domain Sequences

**FIGURE 5:**
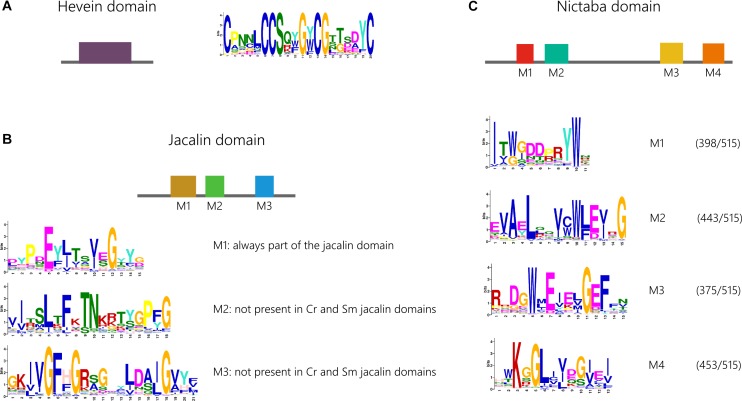
Conservation of functional motifs in the hevein **(A)**, jacalin **(B),** and Nictaba **(C)** domain sequences and corresponding weblogos. Because not all four identified motifs in the Nictaba domain are present in all the analyzed sequences, the number of significant hits for each of the four motifs is indicated. Cr, *Chlamydomonas reinhardtii*; Sm, *Selaginella moellendorffii*.

Motif analysis of the lectin domain sequences for the Nictaba, jacalin and hevein family was performed with MEME to analyze the retention of conserved motifs within the lectin domains across the different lineages (Figure 5). MEME analysis of the hevein domain sequences revealed only one significant motif, shared by sequences from all species under study (Supplementary Table 5). No significant differences were observed between the motif logo made for all sequences compared to the amino acid motif logo made based on the hevein domain sequences from *Arabidopsis thaliana, Oryza sativa, Glycine max* and *Cucumis sativus*. Several cysteine and glycine residues are highly conserved in this motif known to be important for the structure and folding of the hevein domain (Aboitiz et al., 2004). It can be concluded that this motif within the hevein domain is very conserved as it was already part of the hevein domain in both chlorophytes and Phragmoplastophyta.

Analysis of the jacalin domain sequences identified three different motifs, M1-M3 (Figure 5). The order of the three motifs was found to be highly conserved. Further analysis acknowledges motif M1 to be most retained. Moreover, this is the only significant motif that could be identified in jacalin domains from *Chlamydomonas reinhardtii* and *Selaginella moellendorffii*. Jacalin domain sequences from both mosses *Physcomitrella patens* and *Sphagnum fallax* do contain all three motifs, pointing to distinct evolutionary paths. This is again illustrated in the phylogenetic tree in Supplementary Figure 6 in which *Physcomitrella patens* and *Sphagnum fallax* sequences are grouped in separate branches. In gymnosperms and most angiosperms, all three motifs are present.

In the Nictaba domain, four significant motifs (M1-M4) were identified. Most of the sequences contain all four motifs in a specific order (Figure 5). However, there is a considerable number of sequences that only contain three or two motifs. The M2 and M4 motif are retained in 86% and 88% of all sequences, respectively. In contrast, M1 and M2 on the one hand, and M3 and M4 on the other hand, are often both present or absent in sequences that do not contain all four motifs. There is no strong correlation between the origin of the sequence (species) and the preservation of the four motifs. Nor are there significant differences in the sequences of the motif made for all sequences, or for a subset of domain sequences representing the four model angiosperms. Remarkably, the M2 motif is absent in all Polypodiopsida sequences. Except for the M1 motif, all motifs were also retrieved in one or two *Chlamydomonas reinhardtii* sequences and the M4 motif is absent in *Chara braunii*. In the Nictaba domain sequences from the charophyte *Klebsormidium nitens*, all motifs are present with high confidence levels. These data suggest that the M1-M4 motifs originate from an ancestor of Viridiplantae, and that these motifs were not prone to substitution during further evolution.

Several amino acids that were designated to be crucial for the carbohydrate-binding activity of Nictaba, jacalin and hevein lectins, are part of the identified conserved motifs of the lectin domains (the tryptophan residues in M1 for the Nictaba domain; a leucine, tyrosine and aspartic acid residue in M3 of the jacalin domain and the serine and tyrosine residues in the motif identified in the hevein domain). Nevertheless, homologous plant lectins of one particular family can display different carbohydrate-binding specificities (Houlès Astoul et al., 2002; Fouquaert et al., 2009b; Fouquaert and Van Damme, 2012; Agostino et al., 2015). Since no clade-specific motifs were identified in the domain sequences of the Nictaba, jacalin and hevein domain; it is obvious that the conserved amino acids do not act as determinants of carbohydrate-binding specificity. Indeed, it has been reported that the carbohydrate-binding specificity of a lectin domain can change due to amino acid substitutions in loops or sequences which, upon folding of the polypeptide, are located in close vicinity of the binding site. Consequently, different carbohydrate-binding specificities between closely related lectins must result from other determinants that are not part of these motifs or amino acids within a motif at a position that displays sequence variability.

## Conclusion

To increase our understanding of the plant lectin families, we examined the origin of these protein families in the tree of life, with emphasis on the plant lineage. The widespread taxonomical distribution of some plant lectin domains was already described for the GNA, LysM, and ricin B lectin family, while the origin of the jacalin and Nictaba family was revisited. Taken together, our results suggest that different plant lectin families evolved in distinct ways. We documented variations of evolutionary paths at different levels, ranging from horizontal gene transfer, recombination of protein domains and discrepancy in gene loss and duplication events.

The evolution of lectins is characterized by expansion of the different lectin families from algae to higher plants, alongside with the diversification of lectins in terms of domain architecture and possibly functionality. Homologs of most lectin families are also present in extant representatives of charophytes and chlorophytes. Unmistakably, the magnitude of the plant lectin family in rhodophytes, glaucophytes and prasinophytes is far less than that observed in tracheophytes. Only two groups of lectin motifs (LysM and ricin B) have been traced back to glaucophytes/rhodophytes. These are the most abundant plant lectin motifs in Bacteria, indicating that these plant lectin domains are most highly dispersed throughout the tree of life. Many of the essential plant features in land plants have their roots in charophyte algae (Leliaert et al., 2012; Umen, 2014; de Vries and Archibald, 2018; Nishiyama et al., 2018). Several lectin families have been detected in both charophyte algae and Streptophyta, indicating that many lectins originated before the evolution of land plants, and diversified later on. Regarding the domain architecture, an important number of the lectin sequences identified in sisters of vascular plants and streptophytes show resemblances to the domain architecture of animal lectins. It is clear that most of these sequences were not retained during diversification from algae to modern angiosperms. Other lectin domain architectures (e.g., F-box/Nictaba) arose from these ancestral lineages and are conserved in higher plants.

Most lectin sequences encode multi-domain proteins containing at least one lectin domain, suggesting that these proteins exert multiple biological activities. However, it remains challenging to predict the functionality of these lectins based on the domain sequences. Functional studies are needed to better understand their physiological roles. Although our knowledge of plant lectins has increased tremendously, a number of aspects on their evolutionary history remain incompletely understood. In the future, the availability of high quality chromosome-scale assemblies of more (plant) genomes will allow more detailed analyses (Rensing, 2017, 2018). It is apparent that the number of publications addressing the evolution of particular protein families in plants is increasing, and future research will without doubt enhance our understanding of this topic.

## Author Contributions

SVH and EVD outlined and designed the study. SVH performed the research, analyzed the data, and prepared the manuscript. EVD conceived and supervised the study and critically revised the manuscript. All authors have read, revised, and approved the final version of the manuscript.

## Conflict of Interest Statement

The authors declare that the research was conducted in the absence of any commercial or financial relationships that could be construed as a potential conflict of interest.

## Footnotes

1 https://bioinformatics.psb.ugent.be/plaza/versions/plaza_v4_dicots/

2 https://bioinformatics.psb.ugent.be/plaza/versions/gymno-plaza/

3 https://phytozome.jgi.doe.gov

4 http://merolae.biol.s.u-tokyo.ac.jp/blast/blast.html

5 http://cyanophora.rutgers.edu/cyanophora/

6 https://genome.jgi.doe.gov/pages/blast-query.jsf?db=ChlNC64A_1

7 http://www.plantmorphogenesis.bio.titech.ac.jp/~algae_genome_project/klebsormidium/klebsormidium_blast.html

8 http://bioinformatics.psb.ugent.be/blast/moderated/?project=orcae_Chbra

9 https://www.fernbase.org/

10 https://www.ncbi.nlm.nih.gov/Taxonomy/CommonTree/wwwcmt.cgi

11 https://mafft.cbrc.jp/alignment/server/index.html

12 http://tree.bio.ed.ac.uk/software/figtree

13 http://meme-suite.org/
