# Supplementary material for: Messages From the Past: New Insights in Plant Lectin Evolution
Source: Front Plant Sci. 2019 Jan 29;10:36. doi: 10.3389/fpls.2019.00036 (PMC6362431; doi:10.3389/fpls.2019.00036)
Supplement: Supplementary file 1 [file Data_Sheet_1.PDF]

Supplementary Material

Messages From the Past: New Insights in Plant Lectin Evolution

Sofie Van Holle, Els J.M. Van Damme*

*** Correspondence:** Prof dr Els J.M. Van Damme: ElsJM.VanDamme@UGent.be

1 Supplementary Figures and Tables

1.1 Supplementary Figures

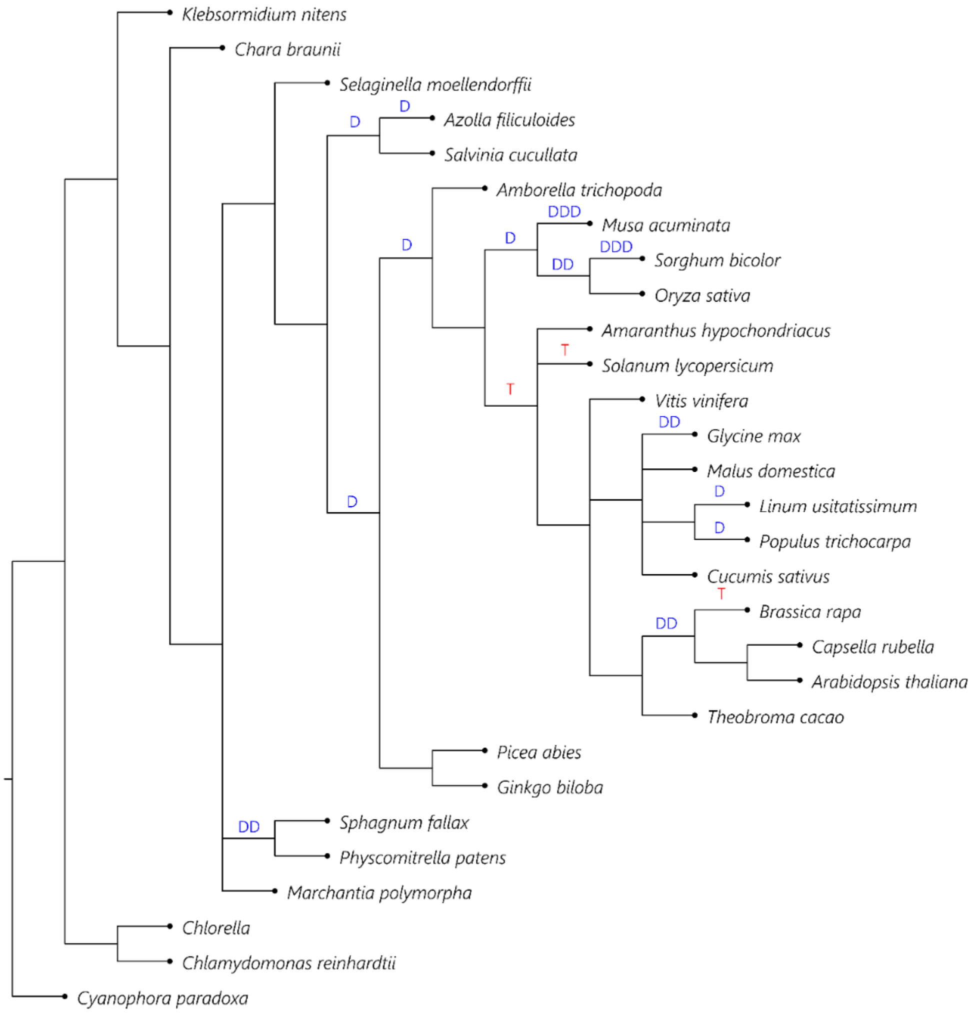

Supplementary Figure 1: Species tree including the 29 completely sequenced plant genomes that were used for reconciliation of the lectin gene trees. Whole genome duplication (D) and triplication (T) events are specified above the specific branches.

Supplementary Figure 2: Reconciliation of the Nictaba tree using Notung 2.9. Red squares (D) denote duplication events and grey colored branches signify lost nodes.

Supplementary Figure 3: Reconciliation of the jacalin tree using Notung 2.9. Red squares (D) denote duplication events and grey colored branches signify lost nodes.

Supplementary Figure 4: Reconciliation of the hevein tree using Notung 2.9. Red squares (D) denote duplication events and grey colored branches signify lost nodes.

Supplementary Figure 5: Maximum likelihood tree constructed with IQ-TREE using Nictaba sequences retrieved from the 29 genomes under study.

Supplementary Figure 6: Maximum likelihood tree constructed with IQ-TREE using jacalin sequences retrieved from the 29 genomes under study.

Supplementary Figure 7: Maximum likelihood tree constructed with IQ-TREE using hevein sequences retrieved from the 29 genomes under study.

1.2 Supplementary Tables

All Supplementary Tables are provided as Excel documents.
