# Supplementary figures and images for: Messages From the Past: New Insights in Plant Lectin Evolution
Source: Front Plant Sci. 2019 Jan 29;10:36. doi: 10.3389/fpls.2019.00036 (PMC6362431; doi:10.3389/fpls.2019.00036)

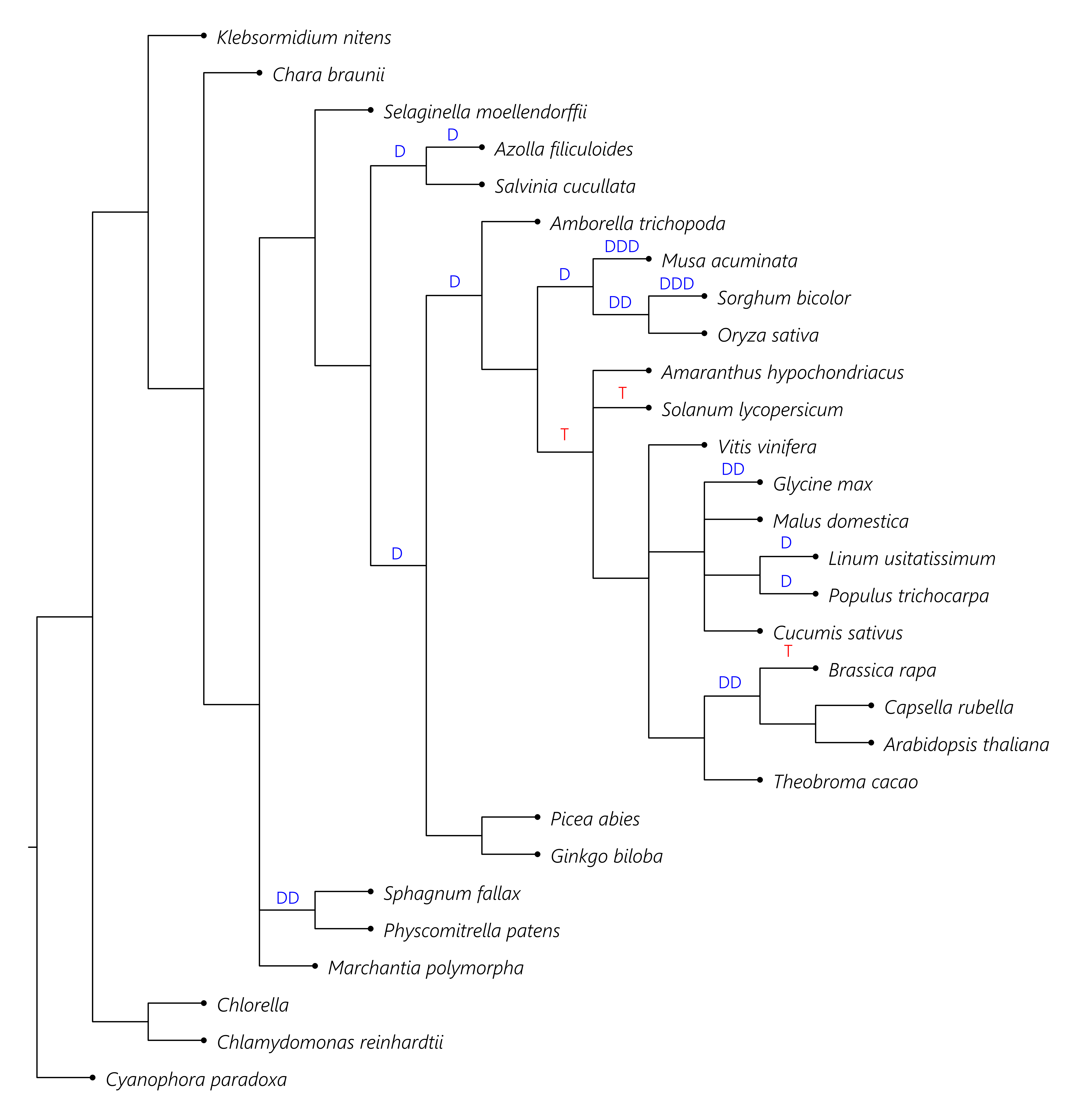

Supplement: Supplementary file 2 [file Image_1.PNG]

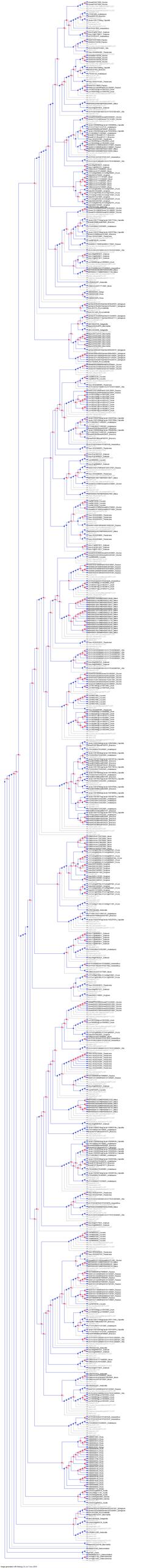

Supplement: Supplementary file 3 [file Image_2.PNG]

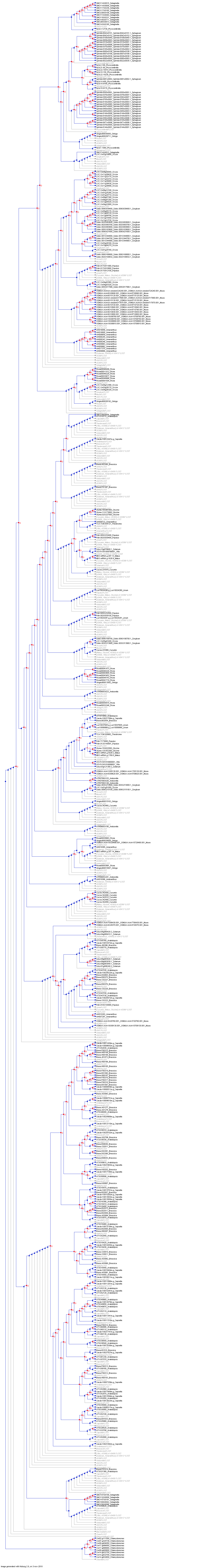

Supplement: Supplementary file 4 [file Image_3.PNG]

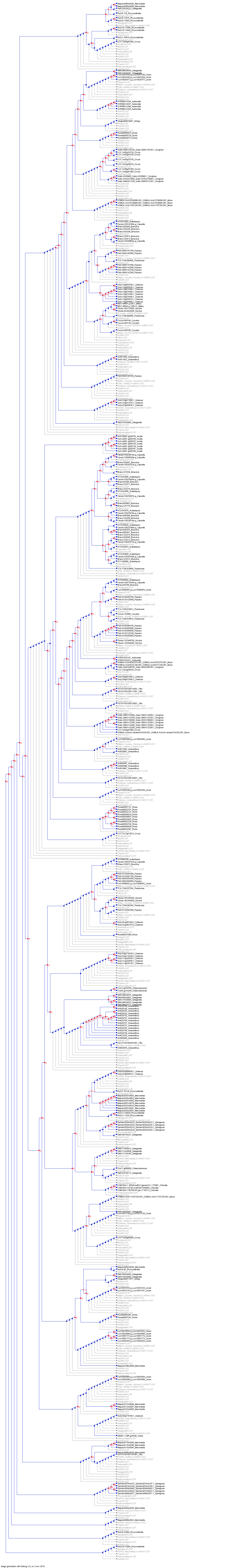

Supplement: Supplementary file 5 [file Image_4.PNG]

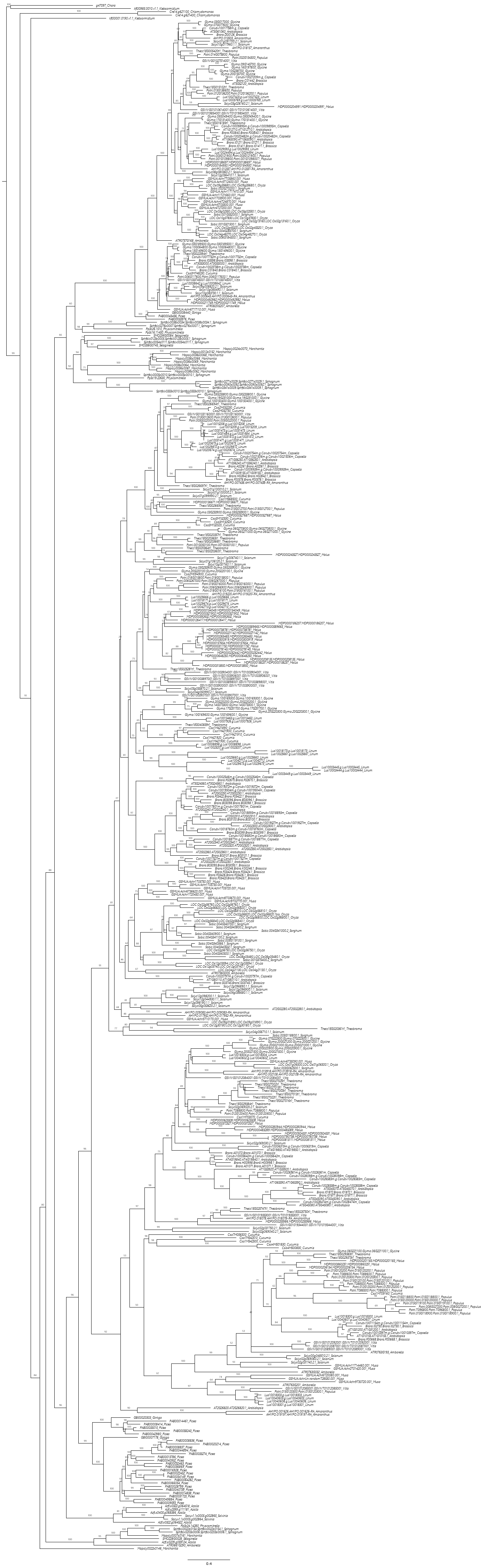

Supplement: Supplementary file 6 [file Image_5.PNG]

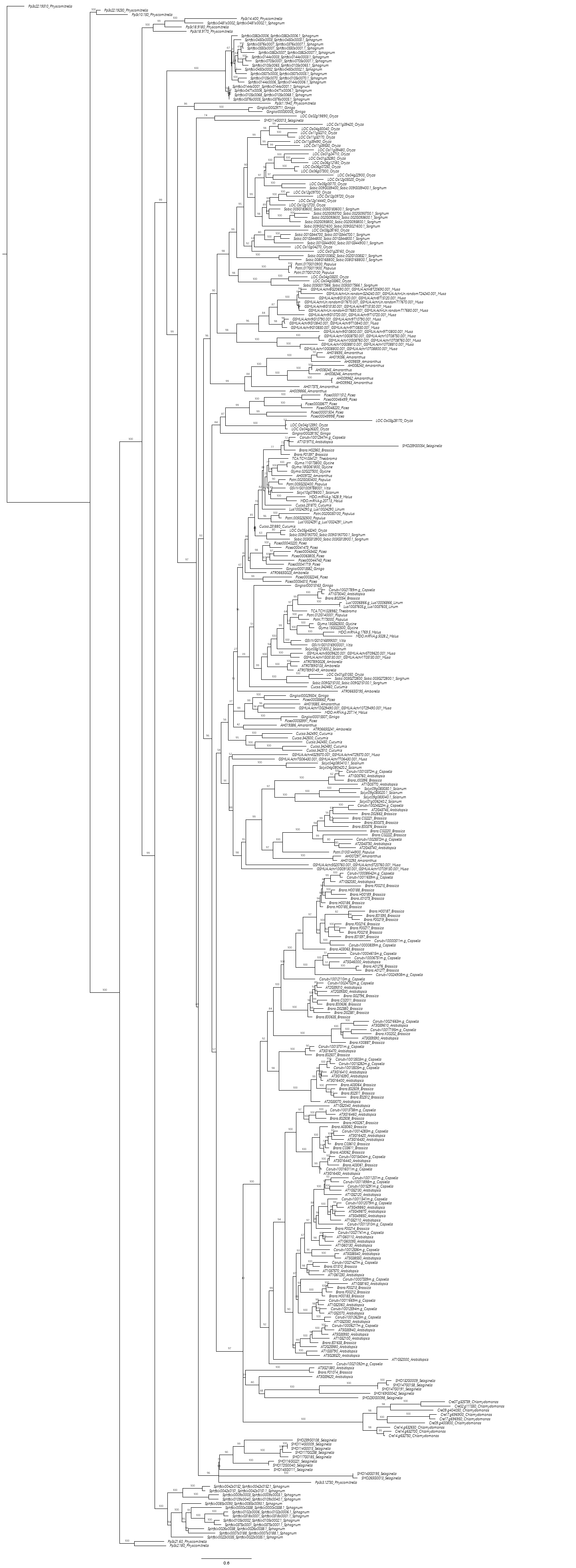

Supplement: Supplementary file 7 [file Image_6.PNG]

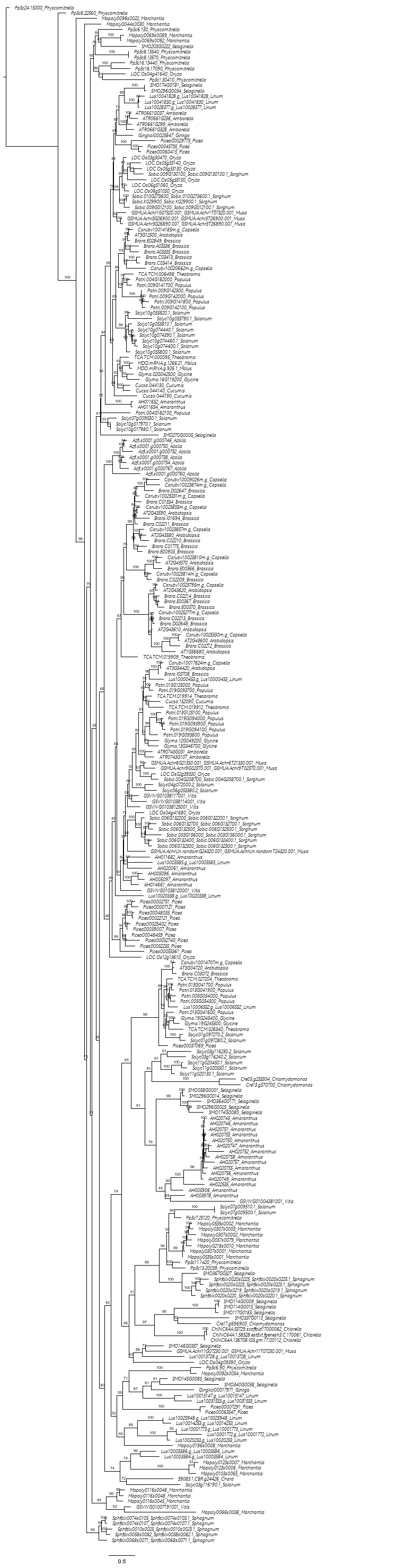

Supplement: Supplementary file 8 [file Image_7.PNG]
